# The effect of 8 weeks of treatment with transcranial pulsed electromagnetic fields on hand tremor and inter-hand coherence in persons with Parkinson’s disease

**DOI:** 10.1186/s12984-019-0491-2

**Published:** 2019-01-31

**Authors:** Anne Sofie Bøgh Malling, Bo Mohr Morberg, Lene Wermuth, Ole Gredal, Per Bech, Bente Rona Jensen

**Affiliations:** 1 Department of Neurology, Odense University Hospital, University of Southern Denmark, Odense, Denmark; 2 Department of Clinical Research, University of Southern Denmark, Odense, Denmark; 3 The Danish Rehabilitation Centre for Neuromuscular Diseases, Taastrup, Denmark; 4 Psychiatric Research Unit, Psychiatric Centre North Zealand, University of Copenhagen, Hillerød, Denmark

**Keywords:** Parkinson’s disease, T-PEMF, PEMF, Rest tremor, Postural tremor, Inter-hand coherence, Accelerometry, Tremor intensity

## Abstract

**Background:**

Parkinson’s disease (PD) tremor comprises asymmetric rest and postural tremor with unilateral onset. Tremor intensity can be amplified by stress and reduced by attention, and the medical treatment is complex. Mirror movements and unintentional synchronization of bimanual movements, possibly caused by insufficient inhibition of inter-hemispheric crosstalk, have been reported in PD, indicating a lag of lateralization.

Potential neuroprotective effects of pulsed electromagnetic fields (PEMF) have been reported in-vitro and in rodents, as have influences of PEMF on human tremor.

The aim was to investigate the effect of 8 weeks daily transcranial PEMF treatment (T-PEMF) of persons with PD on rest and postural hand tremor characteristics and on inter-hand coherence.

**Methods:**

Hand accelerations of 50 PD participants with uni- or bilateral tremor participating in a clinical trial were analysed. A rest and postural tremor task performed during serial subtraction was assessed before and after 8 weeks of T-PEMF (30 min/day, 50 Hz, ±50 V, 3 ms squared pulses) or placebo treatment (sham stimulation 30 min/day). Forty matched healthy persons (no treatment) were included as reference. Intensity and inter-hand coherence related measures were extracted.

**Results:**

The T-PEMF treatment decreased the inter-hand coherence in the PD group with unilateral postural tremor. The PD group with unilateral postural tremor was less clinically affected by the disease than the PD group with bilateral postural tremor. However, no differences between T-PEMF and placebo treatment on either intensity related or coherence related measures were found when all persons with PD were included in the analyses. The peak power decreased and the tremor intensity tended to decrease in both treatment groups.

**Conclusions:**

Eight weeks of T-PEMF treatment decreased inter-hand coherence in the PD group with unilateral postural tremor, while no effects of T-PEMF treatment were found for the entire PD group. The unilateral postural tremor group was less clinically affected than the bilateral postural tremor group, suggesting that early treatment initiation may be beneficial. In theory, a reduced inter-hand coherence could result from a neuronal treatment response increasing inter-hemispheric inhibition. However, this requires further studies to determine. Studies of even longer treatment periods would be of interest.

**Trial registration:**

ClinicalTrials.gov, NCT02125032. Registered 29 April 2014, https://clinicaltrials.gov/ct2/show/NCT02125032?term=NCT02125032&rank=1

**Electronic supplementary material:**

The online version of this article (10.1186/s12984-019-0491-2) contains supplementary material, which is available to authorized users.

## Introduction

Parkinson’s disease (PD) is an asymmetric neurodegenerative disease affecting the dopaminergic neurons especially in the basal ganglia causing cardinal motor symptoms as bradykinesia, tremor, and rigidity. The asymmetric neural degeneration manifests itself as a unilateral onset of motor symptoms followed by a gradual involvement of the contralateral side, although the asymmetric manifestations of motor symptoms persist with disease progression [1].

The classical PD tremor has a main frequency of 4–7 Hz and is most often present at rest in the hands or arms of the affected subjects. Postural and kinetic tremors are also common in the upper extremities whereas tremors in the lower extremities are rarer [2, 3]. Furthermore, re-emergent tremor in terms of tremor re-occurring after repositioning of the limb is frequent [4]. It has been suggested that resting tremor and re-emergent tremor have similar origins as no differences in electromyographic or accelerometric characteristics have been found [5, 6]. In addition, PD tremor intensity can be context-dependent e.g. amplified by stress [7, 8]. The medical treatment of tremor in PD is complex. Thus, the effect of levodopa on tremor can be reduced with cognitive stress [9] and some persons with PD experience levodopa-resistant tremor [10]. Investigation of new treatment methods is therefore warranted.

Mirror movements, where voluntary movements of one limb is accompanied by corresponding involuntary movements of the opposite limb, have been reported in several movement disorders and have been described as a result of inter-hemispheric cross-talk or motor overflow [11, 12]. In accordance, mirror movements have been found to be highly present and persistent in persons with idiopathic PD [13] and to be more pronounced than in healthy peers [14]. Furthermore, persons with PD tend to unintentionally synchronize asynchronic alternating bimanual movements at a lower movement frequency than healthy persons do [15, 16]. The neural origin of such movements might either be uncrossed ipsilateral corticospinal pathways or insufficient inhibition of inter-hemispheric crosstalk, of which the latter is most likely in PD [11, 17]. This phenomenon can be studied by analyzing inter-limb synchronization patterns [12]. Coherence analysis is a standard metric used for studying functional interconnectivity including coupling between body segments in the frequency domain. Inter-hand coherence can be calculated from synchronously measured acceleration of each hand during standardized conditions. Based on the above mentioned literature higher inter-hand coherence in persons with PD compared to healthy controls can be expected.

Treatment with pulsed electromagnetic fields (PEMF) has been suggested to have neuroprotective effects in in-vitro cell-line studies and in-vivo animal studies. For example, PEMF has been shown to enhance cell proliferation and differentiation [18, 19], enhance neurite outgrow [20], regulate neutrophic factors such as BDNF, S100 and NGF [18, 21], stimulate angiogenesis [22], increase microvascular perfusion and tissue oxygenation [23], reduce apoptosis [19], and stimulate neurogenesis in the hippocampal dentate gyrus [24] and in the sub ventricular zone after lesion of substantia nigra [25]. Which molecular mechanisms that are initiated by PEMF are still not fully understood. However, PEMF may affect the tissue both directly through interaction mechanisms between the electromagnetic fields and the conductive tissue, and indirectly by initiating biological events leading to physiologic responses [26]. Recently, we found that 8 weeks of daily treatment with weak transcranial pulsed electromagnetic fields (T-PEMF) improved motor function, in terms of increased rate of force development, in persons with mild PD [27]. Rate of force development depends on the corticospinal drive to the muscles and we proposed that treatment with T-PEMF may increase the corticospinal drive to the muscles through an increased thalamocortical input [27].

To our knowledge, the influence of T-PEMF treatment on PD hand tremor intensity and inter-hand coherence has not previously been studied in a randomized clinical trial. However, a positive influence of weak pulsed electromagnetic fields in the pico-tesla range on tremor intensity in a person with PD has previously been reported in a case report [28]. In addition, extremely low frequent magnetic fields with flux densities in the millitesla-range may be capable of influencing physiologic tremor in healthy subjects shifting the frequency content of physiologic postural tremor towards lower frequencies [29]. This indicates an acute influence of low flux density electromagnetic stimulation on human tremor.

Based on our current knowledge, we hypothesize that repeatedly applied T-PEMF treatment may alter hand tremor characteristics and inter-hand coherence in persons with PD towards normal values measured in healthy controls. Thus, the aim of this study was to investigate the effect of 8 weeks of daily T-PEMF treatment compared to placebo treatment on rest and postural hand tremor characteristics as well as inter-hand coherence in persons with PD.

## Method

### Study design

The present study includes accelerometer data from a subsample of participants of a double-blinded randomized clinical trial [27, 30, 31]. In the trial, 97 participants diagnosed with idiopathic PD (Hoehn & Yahr I-IV) according to the United Kingdom Brain Bank Criteria were randomized to receive either 8 weeks of T-PEMF or placebo treatment in a 1:1 allocation ratio. Inclusion criteria for the trial were an unchanged and optimal medical treatment regarding PD six weeks prior to and during the intervention period; Mini Mental State Examination score > 22; age > 18 years; and cognitive skills enabling certification in the use of the T-PEMF device and to give informed consent. Exclusion criteria were any known neuromuscular or neurological diseases other than PD that might interfere with motor function; psychopathological treatment of other conditions than depression; substance abuse; active medical implants; pregnancy or nursing; current or previous cancer in the brain, head or neck region; leukaemia; autoimmune disease; epilepsy; and open scalp wounds. The clinical trial was registered at clinicaltrials.gov (NCT02125032), was approved by the Regional Scientific Ethical Committees for Southern Denmark (S-20130114) and the Danish Health Authority (CIV-14-01-011780), and was conducted in accordance with the Declaration of Helsinki. All participants provided written informed consent prior to participation. For further details of the trial, please refer to Additional file 1 or [27, 30, 31].

### Participants

For the present study, trial participants diagnosed with idiopathic PD with at least one hand with pathological PD tremor defined by having a rest or postural tremor intensity larger than mean + 2SD of a healthy reference group were included (cut-off values, rest: 0.300 m/s^2^, postural: 0.783 m/s^2^). Fifty of the 97 trial participants with PD had pathological PD tremor (hereinafter referred to as tremor). Seven of these had rest tremor only, 14 had postural tremor only, and 29 had both rest and postural tremor. Thus, 36 participants with PD (17 females, mean age (SD) of 66 (8.9) years, having a total of 52 hands with rest tremor) were included in the analysis of rest tremor, and 43 participants with PD (19 females, mean age (SD) of 65 (9.2), having a total of 64 hands with tremor) were included in the analysis of postural tremor. Endpoint data from participants with a post-interventional treatment compliance of less than 80% were excluded from the analyses. The participants with PD were evaluated by the Unified Parkinson’s Disease Rating Scale (UPDRS) for clinical disease severity characterization [32]. The UPDRS was chosen in preference to the MDS-UPDRS since the latter has not been translated to and validated in a Danish version.

Inspired by our previous finding suggesting that mildly affected persons with PD have a larger potential for neural rehabilitation than more severely affected persons with PD [27], we sub-grouped the participants with PD. Thus, the participants with PD were divided into a group with unilateral tremor and a group with bilateral tremor to investigate the effect of T-PEMF treatment on different levels of disease progression from a tremor perspective.

Additionally, a reference group (REF) of 40 healthy subjects matched on age and sex with no visual detectable tremor (19 females, mean age (SD) of 66 (1.3) years, having a total of 80 non-tremoring hands, Table 1) was included. The REF group was included to provide threshold values for tremor. The REF group was extracted from a larger reference group representing individuals across the adult lifespan (19–83 years) [33]. Initially, the group with ages within the range of the PD group were extracted (*n* = 46). However, the age distribution of the PD group was screwed towards the older ages as expected, whereas the age-range matched reference group was not. Furthermore, women-to-men ratio in the age-range matched reference group was slightly higher than in the PD group. Therefore, six women in the age-range matched reference group were excluded to make the REF group match the PD group on age and sex distribution. This procedure was performed without any knowledge of the reference persons except their age and sex. Group and subgroup baseline descriptive variables are listed in Table 1.

**Table 1:** Group and subgroup description

	Group	N	N, females	Age (years)	Disease duration (years)	LED (mg/day)	UPDRS total	UPDRS motor	UPDRS hand tremor
*PD, rest*									
All	T-PEMF	22	11	68 (6)	6 (5)	547 (439)	49 (15)	28 (9)	3 (2)
	Placebo	14	6	63 (12)	3 (2)	399 (234)	43 (13)	25 (9)	3 (2)
Unilateral	T-PEMF	11	7	69 (5)	5 (5)	562 (470)	41 (12)	23 (8)	2 (1)
	Placebo	9	4	62 (14)	3 (2)	405 (271)	41 (10)	24 (8)	2 (1)
Bilateral	T-PEMF	11	4	67 (7)	7 (5)	531 (427)	57 (12)	33 (8)	5 (2)
	Placebo	5	2	65 (4)	3 (2)	389 (176)	46 (17)	27 (11)	4 (3)
*PD, postural*									
All	T-PEMF	23	10	67 (6)	5 (5)	520 (440)	46 (16)	26 (10)	3 (2)
	Placebo	20	9	63 (11)	4 (3)	474 (346)	46 (14)	26 (9)	3 (2)
Unilateral	T-PEMF	9	5	67 (6)	2 (2)	380 (402)	38 (11)	22 (7)	2 (1)
	Placebo	13	6	62 (12)	4 (3)	436 (264)	43 (13)	25 (8)	2 (1)
Bilateral	T-PEMF	14	5	67 (7)	7 (5)	616 (455)	52 (18)	29 (11)	4 (2)
	Placebo	7	3	63 (10)	4 (2)	545 (480)	50 (18)	29 (11)	3 (3)
*REF*		40	19	66 (8)	–	–	–	–	–

Baseline descriptive variables for Parkinson’s disease (PD) tremor groups, subgroups and the reference group (REF) according to treatment allocation. Disease duration reflects the number of whole years from the time of diagnosis to inclusion. Levodopa equivalent dose (LED) was calculated according to Tomlinson et al. 2010 [43]. The Unified Parkinson’s Disease Rating Scale (UPDRS) hand tremor score reflects the sum of scores for rest and postural hand tremor (item 20.2–3 and 21.1–2). Variables are presented as mean (SD)

### T-PEMF treatment

The T-PEMF treatment has been described in details elsewhere [27, 30, 31]. In short, participants with PD received one 30-min session of home-based T-PEMF or placebo treatment daily for 8 weeks. The T-PEMF device (Re5 NTS Parkinson Treatment System, Re5, Frederiksberg, Denmark) consisted of a pulse generator and a head applicator with 7 electromagnetic coils located as follow: one in the central occipital region, one in the frontal-parietal region (bilateral), and two in the anterior-temporal and posterior-temporal region (bilateral). During T-PEMF treatment, the pulse generator supplied the coils with squared bipolar pulses of ±50 V at 50 Hz and with a pulse duration of 3 ms. The stimulation intensity depends on the distance from the surface of the coil and the radial distance from the centre of the coil. At the periphery of the coil and close to the skull, maximal stimulation was estimated to be 2.5 mV/cm and decreasing with distance [34]. The subjects could not feel this very low stimulation intensity. During placebo treatment, the same treatment duration and treatment device was used but no pulsed electromagnetic fields were generated (sham stimulation). Treatment allocation was encoded to a chip card that was inserted in the pulse generator. The generator interface was identical for the two treatment types. Thus, it was not possible for the participants to see or feel difference between T-PEMF and placebo treatment, and both participants and investigators were blinded to the allocation until after endpoint assessment of the last participant. Participants with PD were optimally medicated at baseline and followed their usual medication scheme throughout the intervention. The REF group did not receive any treatment.

### Protocol and accelerometer measurements

Two-dimensional cylindrical accelerometers (Catsys PD, Danish Product Development Ltd., Snekkersten, Denmark) were fixed on the hand dorsum along and between metacarpal bone II and III on each hand. The accelerometers were sensitive to accelerations in the plane orthogonal to the metacarpal bones. Hand tremor was sampled synchronously from both hands at 50 Hz.

Tremor assessment was performed before (week 0) and within one day after the last treatment session (week 8). Measures at week 0 and 8 were performed on the same time of day for each individual to reduce the influence of intra-day fluctuations. The participants with PD were tested in self-reported ON-state. The subject sat on a chair with backrest and no arm support with their feet on the ground. Hand tremor was assessed in two conditions: 1) rest, while the hands were placed with palms down approximately on the middle of the thigh in a position allowing the subject to relax and 2) postural, while the arms, hands and fingers were extended in front of the body at shoulder height, approximate shoulder width between hands, and with palms facing towards the floor (Additional file 2). PD tremor can be related to the level of attention of the patient [9]. Thus, some patients can deliberately suppress the tremor when focusing on it. Furthermore, PD tremor can be affected by stress with more pronounced tremor in a stressed state [8, 9]. We attempted to standardize the attention level of the participants without inducing stress. Thus, we asked the participants to close their eyes during the assessments to avoid distraction from the environment. Furthermore, we asked the participants to vocally perform a serial subtraction task to focus their attention on the subtraction task and not the tremor task. The use of a serial subtraction task was inspired by Lee et al. [8]. However, we modified the subtraction task and asked the participants to vocally count down from 100 in steps of two (instead of 7 or 8) in a self-paced manner (instead of as quickly as possible) to avoid stress. Finally, before each 30 s assessment, it was emphasized, that the subject should sit as calm and relaxed as possible while counting. The assessments were performed in calm surroundings and two 30-s trials of each condition were performed in the following order: rest, postural, rest, postural. Approximately 30 s pauses separated the assessments.

### Data analysis

Data analyses were performed in MATLAB (Mathworks Inc., USA) using a custom-made script. The script was validated towards conventional tremor analysis software (Catsys PD software, Snekkersten, Denmark). Tremor intensity related measures and inter-hand coherence measures were calculated.

The resultant acceleration was calculated and the 30-s time series were divided into three 10-s time intervals. For each 10-s time interval, the following intensity related measures were calculated within the frequency band of 3–8 Hz (0.1 Hz frequency resolution). Results are presented as the mean across the three time intervals:

- *Tremor intensity* was calculated as $\sqrt{\frac{\sum \limits_{3 Hz}^{8 Hz}{\left| fft\right|}^2}{N^2}}$, where |*fft*| is the absolute value of the fast Fourier transformation of the resultant acceleration in the 3–8 Hz band, and N is the number of |*fft*| observations. According to Parseval’s theorem, this corresponds to the root mean square of the resultant acceleration within the 3–8 Hz frequency band.
- *Peak power* was the maximal absolute power within the 3–8 Hz band.
- *Peak frequency* was the frequency at peak power.

Coherence between two biological time series is a measure of similarity of the power spectra of the signals and high coherence between two signals is interpreted as a high common output from the brain. Inter-hand coherence was calculated on the resulting acceleration concatenated from the two 30-s trials of each condition. We calculated the magnitude squared coherence estimate using Welch’s overlapped averaged periodogram method (window length of 256 samples ~ 5 s, 20% overlap, Hanning window (256 samples)). Three measures in the 3–8 Hz band were extracted:

- *Coherence,* the integral of significant coherence (α = 0.05), being the magnitude squared coherence above a threshold, Z = 0.2058, as described by Terry and Griffin [35, 36] within the 3–8 Hz band.
- *Peak coherence,* the maximal value of magnitude squared coherence within the 3–8 Hz band.
- *Frequency of peak coherence,* the frequency corresponding to peak coherence.

### Statistics

Statistics were performed in SAS 9.4. To investigate potential treatment effects we used linear mixed models for the analysis of the intensity related measures (the log transform of tremor intensity and peak power were used for analyses to meet normal distribution). The analysis of inter-hand coherence related measures was performed by Wilcoxon statistics, as transformation of data did not induce a normal distribution. Age was not correlated to any of the effect measures among the PD group at baseline (Pearson correlation for tremor intensity related measures; Spearman correlation for coherence related measures) and was thus not included as covariate in the models. The level of significance was set to 0.05.

We investigated the effect of treatment on the intensity related measures by the model

$$ dependent\ outcome= week\kern0.17em week\times group $$

with unstructured covariance. *Week* entailed week 0 and week 8, and *group* entailed baseline adjusted treatment groups (T-PEMF and placebo subjects were all considered placebo subjects at week 0). In case of significant *week* × *group* interaction, pairwise comparison within *group* across *weeks* and between *groups* within *week* were performed and a 2-level Bonferroni correction was applied to adjust for multiple comparisons (corrected significance level 0.025). To determine if there was a difference in response to active and placebo treatment on coherence related measures, we used a Wilcoxon Ranked Sum test performed on the differences between week 0 and week 8. The effects of treatment on coherence related measures were evaluated both for the whole PD group and for the uni- and bilateral tremor subgroups.

Intergroup differences between PD and REF on intensity related measures were evaluated by the model *dependent outcome = group*, where group entailed PD and REF and measures from both hands from one subject regarded as repeated measures. To determine differences between participants with PD and REF on the inter-hand coherence measures we used a Wilcoxon Ranked Sum test. Intergroup differences in UPDRS measures were determined by t-test.

Normal distributed variables are presented by means and standard deviations (SD). Non-normal distributed variables are presented by medians and the inter quartile ranges (IQR) indicating where the middle 50% of the data lie.

## Results

### Participants

Of the 36 and 43 participants with PD having rest and postural tremor respectively, three participants with both rest and postural tremor had missing data at week 8 due to withdrawal (*n* = 1) and exclusion from the analyses due to lag of compliance and change of medication (*n* = 2) (all receiving T-PEMF treatment). In addition, one participant with postural tremor only did not show up for endpoint assessment due to personal reasons (placebo). Thus, rest data from 33 subjects (19 T-PEMF and 14 placebo) and postural data from 39 subjects (20 T-PEMF and 10 placebo) were available at week 8. The treatment compliance of the participants completing the intervention was on average 98%.

As expected, the group with unilateral postural tremor was less clinically affected by the disease than the group with bilateral postural tremor as they had a significantly lower UPDRS Total score (unilateral = 41 ± 12, bilateral = 51 ± 17, *p* = 0.0361) and a tendency of lower UPDRS Motor score (unilateral = 24 ± 8, bilateral = 29 ± 11, *p* = 0.0590). Thus, the subgroup with bilateral tremor represented a clinically more severely affected group than the subgroup with unilateral tremor. The subgroups with uni- and bilateral rest tremor consisted of 19 participants (10 T-PEMF, 9 placebo) and 13 participant (9 T-PEMF, 5 placebo) respectively. The subgroups with uni- and bilateral postural tremor consisted of 20 participants (8 T-PEMF, 12 placebo) and 19 participants (12 T-PEMF, 7 placebo) respectively.

The adverse events of the treatments have been reported elsewhere. They were benign, mild and transient and the frequency did not differ between treatment groups [31].

### PD vs REF

**Fig. 1:**
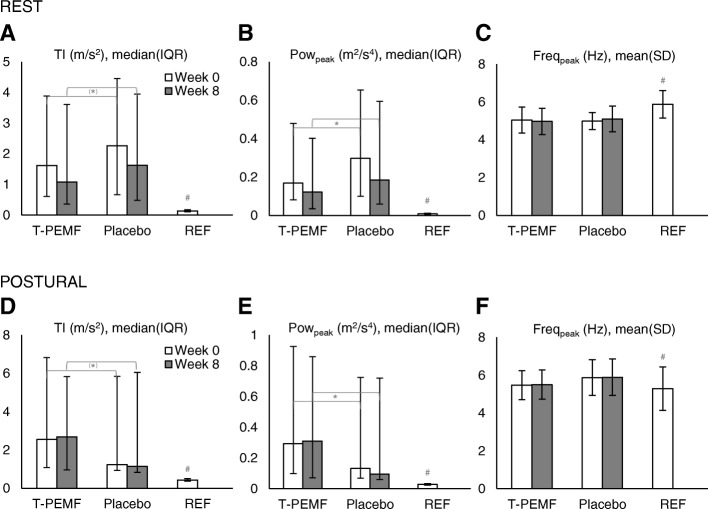
Treatment effect on intensity related measures for the rest and postural condition, measured values. **A** and **D** Tremor intensity (TI). **B** and **E** Peak power (Pow_peak_). **C** and **F** Peak power (Freq_peak_). T-PEMF = transcranial pulsed electromagnetic fields. REF = healthy reference group. * = significant difference from week 0 to week 8 across treatment groups (*P* ≤ 0.05). ^(^*^)^ = tendency of difference from week 0 to week 8 across treatment groups (0.05 < *P* ≤ 0.1). ^#^ = significant difference between the treatment groups combined at week 0 and REF (*P* ≤ 0.05)

**Fig. 2:**
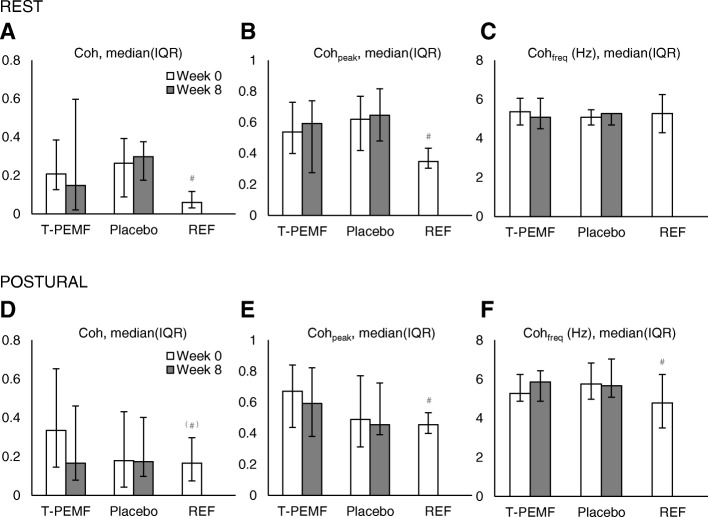
Treatment effect on inter-hand coherence measures for the rest and postural condition, measured values. **A** and **D** Coherence (Coh). **B** and **E** Peak coherence (Coh_peak_). **C** and **F** Frequency of peak coherence (Coh_freq_). T-PEMF = transcranial pulsed electromagnetic fields. REF = healthy reference group. * = significant difference from week 0 to week 8 across treatment groups (*P* ≤ 0.05). ^#^ = significant difference between the treatment groups combined at week 0 and REF (*P* ≤ 0.05). ^(#)^ = tendency of difference between the treatment groups combined at week 0 and REF (0.05 < *P* ≤ 0.1)

The PD group had a much larger median tremor intensity (rest: PD 1330% of REF, *p* < 0.0001, Fig. 1A; postural: PD 445% of REF, *p* < 0.0001, Fig. 1D) and median peak power (rest: PD 2336% of REF, *p* < 0.0001, Fig. 1B; postural: PD 770% of REF, *p* < 0.0001, Fig. 1E) than the REF group in both conditions. In addition, the PD group had slightly lower estimated mean peak frequency at rest (PD 85% of REF, *p* < 0.0001, Fig. 1C) but a slightly higher peak frequency than the REF group at the postural condition (PD 107% of REF, *p* = 0.0421, Fig. 1F). We found a higher median coherence (rest: PD 384% of REF, *p* < 0.0001, Fig. 2A; postural: PD 185% of REF, *p* = 0.0704, Fig. 2D), median peak coherence (rest: PD 165% of REF, *p* < 0.0001, Fig. 2B; postural: PD 130% of REF, *p* = 0.0271, Fig. 2E), and median frequency of peak coherence (postural: PD 114% of REF, *p* = 0.0339, Fig. 2F) in the PD group compared to REF in both conditions, except for the frequency of peak coherence at rest where no between group difference was found (*p* = 0.7784, Fig. 2C). When dividing into the uni- and bilateral tremor subgroups, both subgroups had significantly higher median coherence (unilateral: 0.233 ≈ 384% of REF, *p* < 0.0001; bilateral; 0.277 ≈ 456% of REF, *p* = 0.0010) and median peak coherence (unilateral: 0.620 ≈ 178% of REF, *p* < 0.0001; bilateral; 0.555 ≈ 159% of REF, *p* = 0.0011) than REF in the rest condition. However, in the postural condition only the bilateral tremor group differed from REF (coherence: 0.416 ≈ 251% of REF, *p* = 0.0042; peak coherence: 0.666 ≈ 46%, *p* = 0.0097).

### Effect of the T-PEMF treatment

The statistical analysis showed no statistical difference between the effect of T-PEMF and placebo treatment on tremor intensity, peak power or peak frequency. However, main effects of time across treatment groups were found. Thus, the tremor intensity tended to decrease from week 0 to week 8 in both treatment groups in the resting (*p* = 0.0604, model estimated change in median for each group: T-PEMF -22%, placebo − 23%, Fig. 1A) and in the postural condition (*p* = 0.0585, model estimated change in median for each group: T-PEMF -3%, placebo − 19%, Fig. 1D), but no difference of improvement between groups was found. Likewise, peak power decreased significantly from week 0 to week 8 across groups (rest: *p* = 0.0453, model estimated change in median for each group: T-PEMF -32%, placebo − 22%, Fig. 1B; postural: *p* = 0.0128, model estimated change in median for each group: T-PEMF -7%, placebo − 28%, Fig. 1E), but no difference of improvement between groups was found. The peak frequency did not change (Fig. 1C & F).

With all participants included, no statistical differences in coherence, peak coherence or frequency of peak coherence were found between T-PEMF and placebo treatment (Fig. 2). However, when rerunning the analysis after sub-grouping into unilateral and bilateral tremor groups, a statistically significant treatment effect was found for the PD group with unilateral tremor in the postural condition (*p* = 0.0339). Thus, coherence was reduced in the T-PEMF group with unilateral postural tremor (Δcoherence from week 0 to 8, median (IQR): − 0.10 (− 0.190 to − 0.0019)) while the corresponding coherence values for the placebo group was not (Δcoherence from week 0 to 8, median (IQR): + 0.068 (− 0.052 to + 0.147)).

## Discussion

A major finding of the study was that an 8-week T-PEMF treatment decreased the inter-hand coherence in the PD group with unilateral postural tremor. The PD group with unilateral postural tremor was less clinically affected by the disease than the PD group with bilateral postural tremor. However, no differences between T-PEMF and placebo treatment on either intensity related or coherence related measures were found when all persons with PD were included in the analyses. The peak power decreased and the tremor intensity tended to decrease across treatment groups.

### Effect of T-PEMF on inter-hand coherence

A major new finding was that eight weeks of treatment with T-PEMF plausibly reduced coherence relative to placebo treatment among the participants with unilateral postural tremor. Thus, T-PEMF seemed to lower the common input to movement-patterns of the limbs within the PD frequency range (3–8 Hz). Interestingly, the group having unilateral postural tremor was less clinically affected by the disease evaluated by UPDRS Total and Motor scores. This pattern of the T-PEMF treatment positively affecting a less affected group distinctively from placebo treatment was also found in the analysis of the influence of T-PEMF treatment on functional rate of force development in the clinical trial. Here, the least functionally impaired PD group benefitted from the T-PEMF treatment relative to placebo treatment by increasing their functional rate of force development, while the most functionally impaired PD group did not [27]. A recent study on repetitive transcranial magnetic stimulation in PD rats showed that functional dopaminergic neurons in substantia nigra are required to induce motor plasticity [37]. Thus, a certain level of neural functionality may need to be present to gain positive effects of neurostimulation. This emphasizes the importance of early initiation of daily treatment with T-PEMF in PD.

A possible neural explanation for the positive effect of T-PEMF in the unilateral postural tremor group (least affected) could be an increased inhibition of the inter-hemispheric crosstalk [11, 17].

Extending the treatment period could potentially induce a larger effect, and could maybe induce effects in more affected persons with PD as well. However, this requires further investigations to determine.

### Effect of treatments on intensity related measures

Both T-PEMF and placebo treatment reduced peak power and tended to reduce tremor intensity in both the postural and resting condition. However, the T-PEMF group did not differ statistically from the placebo group. We attempted to induce a calm environment during the assessments, as stress is known to amplify tremor in PD [8, 9]. However, we cannot exclude the possibility that the participants experienced less stress post treatment due to the familiarity of the test situation. Furthermore, previous studies have reported significant placebo effects on PD tremor. A recent study of the effect of placebo on resting tremor in persons with PD showed a major reduction of tremor amplitude 30 min after a subcutaneous injection of saline, which they were told was apomorphine, a dopamine agonist. This effect was found in approximately half of the subjects classified as placebo responders who benefitted equally from a saline and an apomorphine injection [38]. Thus, using tremor intensity as an effect measure in clinical trials is challenging, as it is highly sensitive to other factors such as stress, level of attention, and placebo effects and thereby may mask a potential treatment effect. Thus, we cannot exclude the possibility of an effect of 8-weeks of T-PEMF treatment on tremor intensity and peak power, but we can conclude that the group effect of the T-PEMF treatment was not statistically different from the effect of the placebo treatment.

### Inter-hand coherence PD vs. REF

Our results showed that inter-hand coherence and peak coherence were larger in the PD group than in the REF group. At first, this seems to be in contrast to a recent finding by Morrison et al. of no difference of peak coherence of postural hand tremor accelerations between persons with PD having bilateral tremor and healthy peers [39]. However, this discrepancy may be caused by analytic differences, as our analyses were specified to the parkinsonian tremor band (4–7 Hz extended by 1 Hz to 3–8 Hz) to focus the analysis. The study by Morrison et al. included tremor frequencies up to 40 Hz and thus included frequencies of both the parkinsonian and physiological tremor bands. Morrison et al. found that 75% of the accumulated proportional tremor power in the healthy group extended up to 10–11 Hz while it was found below 5–6 Hz in the PD group [39]. In addition, they found the mean frequency of peak coherence for the healthy group to be 8.9 Hz. Thus, when focussing on tremor in the 3–8 Hz band in our study, we by-passed most of the physiological tremor band and may not have included the frequencies of largest coherence in the REF group. However, the peak coherence values found by Morrison et al. (2012) were lower for both the PD and healthy peers (both group means around 0.2), than the values of the present study. The findings of higher peak coherence in the present study during both rest and postural conditions may be attributed to the attention distracting counting task. Performing attention distracting subtraction tasks has been shown to increase intra-limb inter-muscular coherence at rest in persons with PD [40], and to decrease the complexity (i.e. lower the sample entropy) of an EMG signal while performing an isometric contraction in healthy subjects [41]. In addition, inter-hemispheric connectivity has been shown to increase with the addition of a counting task to a unimanual tapping task, though this increase was not significant during a bimanual tapping task [42]. Together, this indicates that performing a task while distracting attention gives rise to a more synchronized motor unit activation that could plausibly occur bilaterally and thus affect inter-hand coherence.

In addition, it is an interesting finding that coherence was higher in the PD group with unilateral rest tremor than in healthy peers, which suggests a more similar frequency content of hand movements between hands during the resting condition in the PD group with unilateral tremor. This indicates that the lag of relaxation and/or control of movement are bilaterally affected despite the fact that only unilateral tremor was present.

### Methodologic considerations

In participants with substantial tremor, there is a risk of mechanical transmission of tremor from leg to hand and even from one hand to the other especially during the resting condition. However, in the present randomized study we expect both treatment groups to be equally affected.

## Conclusion

Our findings revealed that eight weeks of T-PEMF treatment decreased the inter-hand coherence in the PD group with unilateral tremor, while no effects of T-PEMF treatment were found for the entire PD group. The unilateral postural tremor group was less clinically affected by the disease than the bilateral postural tremor group, which suggests that early treatment initiation may be beneficial. In theory, a reduced inter-hand coherence could be a result of a neuronal treatment response increasing inter-hemispheric inhibition. However, this requires further studies to determine. Likewise, it would be of interest to explore if even longer treatment period would enhance the treatment effect.

## Additional files

Additional file 1: Specifications of the clinical trial. (DOCX 19 kb)

Additional file 2: Pictures of tremor assessments. (PDF 319 kb)

## Abbreviations

IQR: Inter-Quartile Range
PD: Parkinson’s disease
PEMF: Pulsed electromagnetic fields
REF: Healthy reference participants
T-PEMF: Transcranial Pulsed Electromagnetic Fields
