# Supplementary material for: The effect of 8 weeks of treatment with transcranial pulsed electromagnetic fields on hand tremor and inter-hand coherence in persons with Parkinson’s disease
Source: J Neuroeng Rehabil. 2019 Jan 31;16:19. doi: 10.1186/s12984-019-0491-2 (PMC6357382; doi:10.1186/s12984-019-0491-2)
Supplement: Supplementary file 1 — Specifications of the clinical trial. (DOCX 19 kb) [file 12984_2019_491_MOESM1_ESM.docx]

Additional file 1

**Specifications of the clinical trial**

*Sample size estimation*

We conducted a randomized clinical trial with various outcome measures. We chose the Unified Parkinson’s Disease Rating Scale (UPDRS) total score for sample size calculation and considered it a continuous response variable. We wanted two equal sized treatment groups (active and placebo) and to asses them pre and post treatment. Prior data indicate, that the minimal clinically important change of the UPDRS total score is 3 points (SD of change = 7, corresponding to an effect size of 0.43) [1, 2]. To be able to reject the null hypothesis that a true change in mean of 3 points from baseline to endpoint in the active or placebo group with probability (power) 0.8, we would need a group size of 45 subjects. Thus, a sample size of 90 subjects were estimated to be sufficient (45 subjects receiving active and 45 receiving placebo treatment). The Type I error probability associated with the paired t-test test of this null hypothesis is 0.05.

*Settings and locations*

Inclusion was performed in Odense University Hospital, Denmark, and in a facility located in Taastrup, Denmark, by the investigators. All assessments were performed in a movement laboratory located in Taastrup, Denmark.

*Recruitment*

Participants with Parkinson’s disease (PD) were recruited from Odense University Hospital and private neurologists in Denmark from May 2014 to August 2015. The healthy participants were included from Dec 2015 to May 2016 as a reference group and were recruited among spouses of participants with PD and by a convenience sample of local residents.

*Randomization*

Included participants with PD were randomized to receive 8 weeks of T-PEMF or placebo treatment. The T-PEMF and placebo group were of equal size. A third party person conducted the allocation. Participants with PD were allocated to a number on the allocation list in order of inclusion. Chip cards were numbered and encoded by the third party persons in accordance to allocation list. The chip cards determined if the T-PEMF device produced T-PEMF treatment or placebo treatment (sham stimulation). The allocation list was not revealed to the participants or investigators until after the post-assessment of the last participant.

*Participant flow*

137 persons with PD were assessed for eligibility for the clinical trial. 40 did not meet inclusion criteria, thus 97 participants with PD were included and initiated treatment (49 T-PEMF, 48 placebo). Three did not finish the intervention (2 withdrawals, 1 exclusion due to hospitalization nonrelated to the trial, all received T-PEMF treatment) [3]. From the 94 completing the intervention, endpoint data of three were excluded due to lag of compliance and change of medication. Of the remaining 91 participants with PD, 36 and 43 had rest and postural tremor at week 0, respectively, and were included in the present study.

1. Schrag A, Sampaio C, Counsell N, Poewe W. Minimal clinically important change on the unified Parkinson's disease rating scale. Movement disorders : official journal of the Movement Disorder Society. 2006;21(8):1200-7. Epub 2006/05/05. doi: 10.1002/mds.20914. PubMed PMID: 16673410.

2. Hauser RA, Auinger P. Determination of minimal clinically important change in early and advanced Parkinson's disease. Movement disorders : official journal of the Movement Disorder Society. 2011;26(5):813-8. Epub 2011/03/26. doi: 10.1002/mds.23638. PubMed PMID: 21437987.

3. Morberg BM, Malling AS, Jensen BR, Gredal O, Bech P, Wermuth L. The Effects of Transcranial Pulsed Electromagnetic Field stimulation on quality of life in Parkinson's Disease. Eur J Neurol. 2018. doi: 10.1111/ene.13637. PubMed PMID: 29573167.
