# Supplementary figures and images for: The effect of 8 weeks of treatment with transcranial pulsed electromagnetic fields on hand tremor and inter-hand coherence in persons with Parkinson’s disease
Source: J Neuroeng Rehabil. 2019 Jan 31;16:19. doi: 10.1186/s12984-019-0491-2 (PMC6357382; doi:10.1186/s12984-019-0491-2)

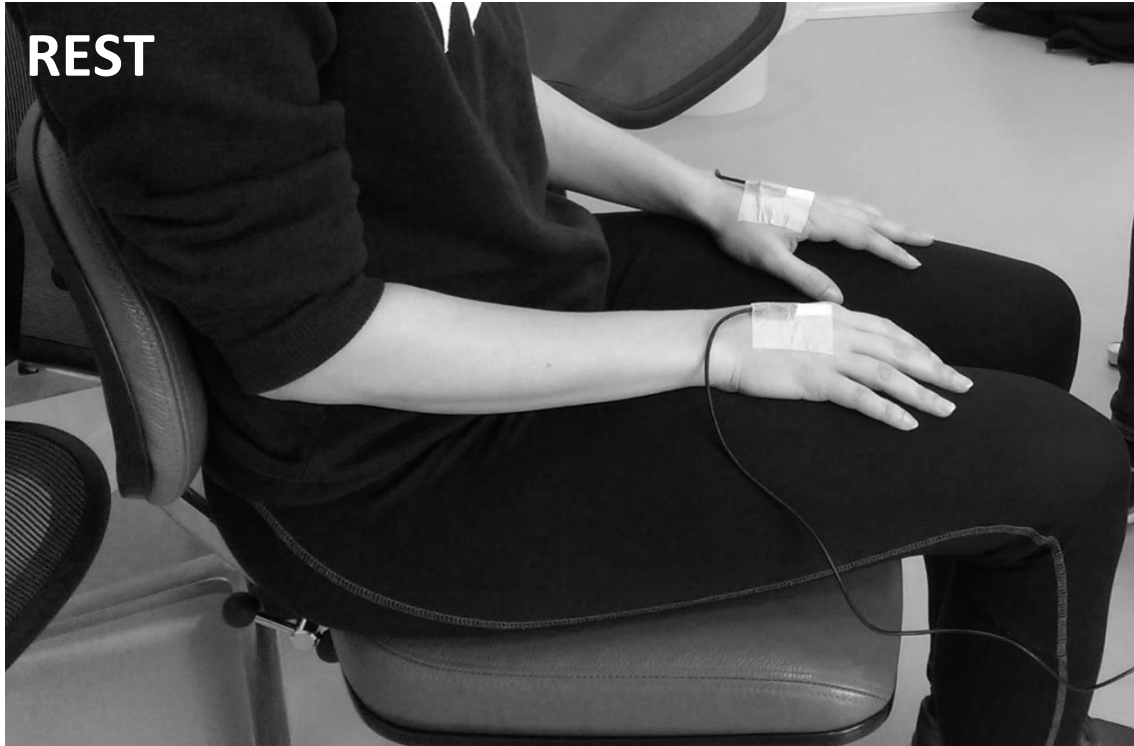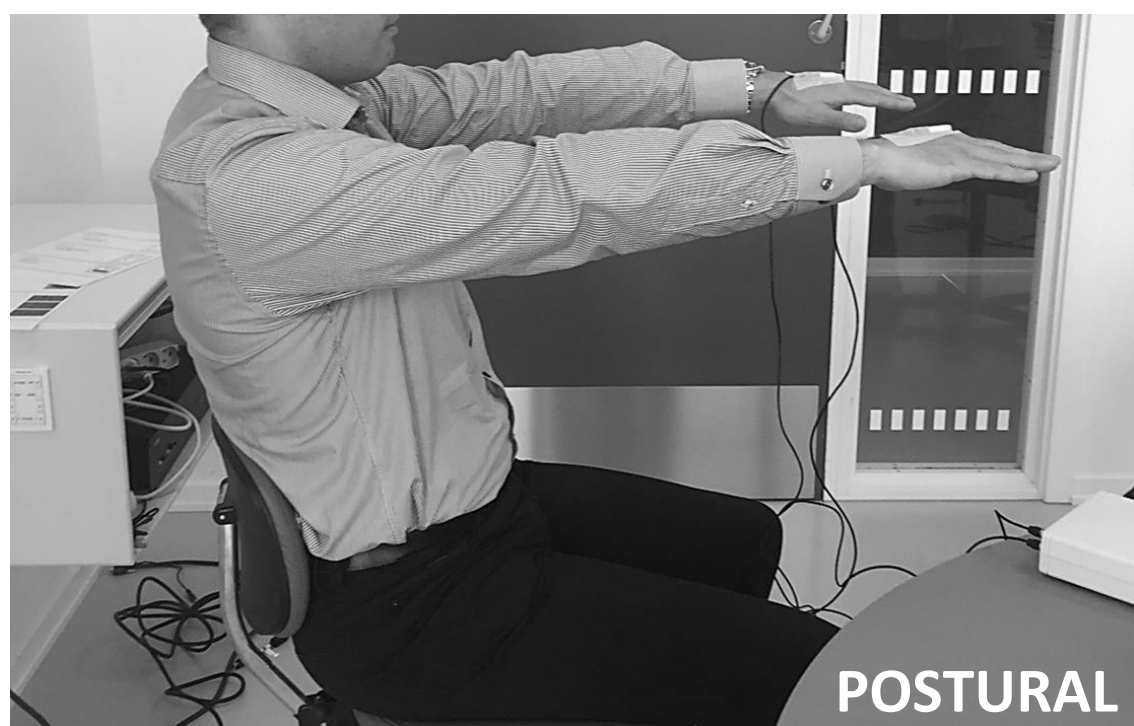

Supplement: Supplementary file 2 — Pictures of tremor assessments. (PDF 319 kb) [file 12984_2019_491_MOESM2_ESM.pdf]
